# Where Does Auto-Segmentation for Brain Metastases Radiosurgery Stand Today?

**DOI:** 10.3390/bioengineering11050454

**Published:** 2024-05-03

**Authors:** Matthew Kim, Jen-Yeu Wang, Weiguo Lu, Hao Jiang, Strahinja Stojadinovic, Zabi Wardak, Tu Dan, Robert Timmerman, Lei Wang, Cynthia Chuang, Gregory Szalkowski, Lianli Liu, Erqi Pollom, Elham Rahimy, Scott Soltys, Mingli Chen, Xuejun Gu

**Affiliations:** 1 Department of Radiation Oncology, Stanford University, Stanford, CA 94305, USA; 2 Department of Radiation Oncology, UT Southwestern Medical Center, Dallas, TX 75390, USA; 3 NeuralRad LLC, Madison, WI 53717, USA

**Keywords:** brain metastases (BMs), segmentation, deep learning

## Abstract

Detection and segmentation of brain metastases (BMs) play a pivotal role in diagnosis, treatment planning, and follow-up evaluations for effective BM management. Given the rising prevalence of BM cases and its predominantly multiple onsets, automated segmentation is becoming necessary in stereotactic radiosurgery. It not only alleviates the clinician’s manual workload and improves clinical workflow efficiency but also ensures treatment safety, ultimately improving patient care. Recent strides in machine learning, particularly in deep learning (DL), have revolutionized medical image segmentation, achieving state-of-the-art results. This review aims to analyze auto-segmentation strategies, characterize the utilized data, and assess the performance of cutting-edge BM segmentation methodologies. Additionally, we delve into the challenges confronting BM segmentation and share insights gleaned from our algorithmic and clinical implementation experiences.

## 1. Introduction

Brain metastases (BMs) manifest in approximately 20% of all cancer patients, especially for lung, breast, renal, and melanoma type cancers [1], and the incidence is increasing as systemic therapies advance and patients live longer [2]. Historically, whole-brain radiotherapy (WBRT) was the standard of care for BMs. However, due to the cognitive impairment caused by WBRT, stereotactic radiosurgery (SRS) has gained prominence and emerged as a favored regimen for patients with a limited number of BMs [3,4,5,6].

SRS is a precision irradiation technique designed to target small lesions with an ablative dose and is usually planned using contrast-enhanced T1-weighted magnetic resonance imaging (T1c-MRI) for identifying and delineating BM regions. While SRS has shown significant efficacy in treating BMs and adherence to critical radiation dose thresholds for patient safety [7,8,9], achieving optimal local control and minimizing damage to normal brain tissue necessitate accurate BM detection and precise segmentation.

**Figure 1:**
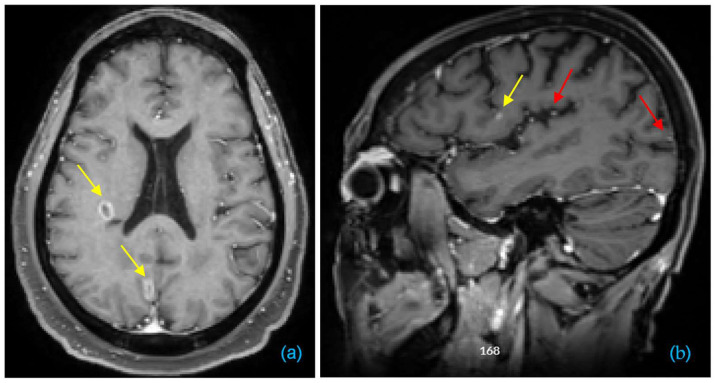
Examples of T1c showing two BM cases. (**a**) Axial view showing two ring-enhancing BMs indicated by yellow arrows. (**b**) Sagittal view showing a small BM indicated by a yellow arrow, alongside examples of blood vessels indicated by red arrows.

However, detection and segmentation of BMs pose substantial challenges due to the heterogeneity among BM patients. This heterogeneity includes variations in BM volume, the number of BMs per patient, nodular or ring-enhancing patterns (as illustrated in Figure 1a), and diverse locations. Additionally, subtle differences, such as the extent of peritumoral edema and necrosis, as well as the microvasculature appearance of BM, may complicate the boundary. Despite exhibiting hyperintensity on T1c-MRI, BMs’ inconspicuous nature—especially when small and resembling blood vessels, as illustrated in Figure 1b—and the potential for them to be numerous add unique detection and segmentation challenges compared to other tumors. Current clinical practice involves manual detection of BMs, which is time-consuming and subject to observer variability. This may lead to BMs remaining undetected unless identified in subsequent follow-up imaging [10,11].

Several studies have proposed automated methods for BM detection and segmentation, leveraging computer-aided detection (CAD) techniques like template matching, active contouring, and support vector machines [12,13,14,15,16]. While these approaches have shown promise, challenges remain in ensuring their robustness [17]. In recent years, the advent of machine learning, particularly deep learning, has enabled the automation of BM delineation in SRS.

Numerous reports have highlighted the use of deep learning for brain tumor segmentations [18,19], and algorithms developed for other brain tumors, like glioma, are often adaptable for segmenting large BMs [20]. However, when it comes to smaller BMs, the existing delineation algorithms tend to exhibit poorer performance [20]. Yet there is limited literature that specifically differentiates or focuses on small BMs less than 1 cm in diameter [21,22]. Rather, studies emphasize BM delineation strategies that target all BM sizes, which consequently can positively and negatively influence the efficacy of these automation methods.

This paper sets out to review the current state-of-the-art BM auto-segmentation. It explores segmentation strategies, data utilization, and segmentation accuracy, discusses challenges confronting BM segmentation, and offers insights from our algorithmic and clinical implementation experience.

## 2. Studies Included in Our Review

A literature search was conducted on PubMed for publications between 2015 and 2023. The search was limited to English-language articles using a set of keywords related to automated identification of BMs: ((automated) OR (automatic)) AND ((machine learning) OR (deep learning)) AND ((segmentation) OR (detection)) AND (brain) AND (metastases). Following initial screening and supplemented by additional articles identified through cross-referencing, a total of 19 studies were included in our review (Table 1).

All studies used T1c-MRI, and about half of the studies used more than one sequence. For multiparametric sequences, co-registration was applied in preprocessing. Most studies applied preprocessing, including skull stripping, resampling, and intensity normalization. Below, we give an overview of the strategies, data utilized, and results in each study.

Liu et al. improved DeepMedic [42] with an added convolutional neural network (CNN) path featuring 5 × 5 × 5 kernels, creating enDeepMedic, to capture features at additional scales for BM segmentation. They benchmarked the model utilizing multiparametric glioma data (265 cases) from the Brain Tumor Segmentation (BraTS) 2015 Challenge and obtained favorable comparisons with the Challenge results. When trained and evaluated on their institutional dataset of BM cases (225 cases) with T1c MRI, the model achieved an overall dice similarity coefficient (DSC) of 0.67 ± 0.03, a mean surface-to-surface distance of 0.9 ± 0.3 mm, and a standard deviation of surface-to-surface distance of 0.8 ± 0.1 mm across all BM volumes [23].

Charron et al. investigated the use of single- and multi-modality MRI sequences (T1, T1c, and FLAIR) for BM segmentation employing DeepMedic [42]. They implemented data augmentation, termed as virtual patients, to enhance the model. Utilizing T1c in combination with FLAIR resulted in slightly improved performance (DSC of 0.78, sensitivity of 0.97, and false positive rate (FPR) of 5.9 per patient) compared to T1c alone, while incorporating all three sequences yielded the lowest sensitivity, albeit marginally. Furthermore, they explored segmentation with three output channels i.e., background, necrotic region, and enhanced region, observing a slight enhancement in detection sensitivity, which is potentially attributed to a proportionally increased weighting for the lesions [24].

Hu et al. utilized multi-modality images, including T1c-MRI and CT, with resolutions of 0.6 mm × 0.6 mm × 2 mm. Their data preprocessing involved slice-wise adaptive histogram equalization and volume-wise z-score normalization for intensity adjustment. For BM detection and segmentation, they employed ensemble deep neural networks, specifically 3D U-Net and DeepMedic. Addressing data imbalance, they applied focal dice loss (volume-aware) and found that batch re-weighting outperformed other weighting schemes. During training, they imposed a sampling criterion, ensuring BM was present in more than 70% of the data. During testing, the model was evaluated separately for two groups of BMs based on volume—less than or greater than 1500 mm^3^. The results showed detection sensitivity of 0.61 and 0.98 and dice scores of 0.47 and 0.82, respectively [25].

Dikici et al. focused on the detection of BM with a size less than 15 mm, utilizing 3D T1c-MRI. Their approach involved applying a blob filter, specifically a Laplace operator followed by Gaussian blurring, to extract candidate positions. Subsequently, a small volume (16 × 16 × 16 mm^3^) centered at each candidate position was cropped out as input of a 3D CNN for BM classification. To mitigate data imbalance, positive and negative classes were paired, each constituting 50% during training. Additionally, data augmentation techniques, including rotation, deformation, and intensity adjustment, were employed. The study reported an overall sensitivity of 90% with a false positive rate of 9.12 per patient [26].

Grovik et al. adapted GoogLeNet by omitting the first and third down-sampling layers to reduce the down-sampling rate and added a deconvolutional layer at the end for BM segmentation. They used input images of 2.5D, encompassing ±3 slices around each center slice to capture through-plane features. The input images comprised four MRI sequences, i.e., Pre/Post 3D T1 CUBE, 3D T1c BRAVO, and 3D CUBE FLAIR, and the data were preprocessed, including skull stripping. Their focus was on BMs larger than 10 mm^3^. The model achieved a DSC of 0.79 ± 0.12, precision of 0.79 ± 0.12, and recall of 0.53 ± 0.22 [27].

Xue et al. employed cascaded fully convolutional networks (FCNs), referred to as BMDS, for BM detection followed by segmentation. The detection network with FCN produces a classification map with reduced resolution compared to the input MRI. This lower-resolution classification map is utilized to generate bounding boxes for the second-stage segmentation on the image of the original resolution. The authors utilized post-contrast 3D T1 magnetization-prepared rapid acquisition gradient echo (MPRAGE) and included intensity normalization in their data preprocessing, although skull removal or registration was not performed. They grouped BMs by size (6–18 mm and 18–45 mm) and evaluated the model separately for each size group (DSC 0.83 and 0.89, respectively) [28].

Bousabarah et al. utilized multiparametric images, including T1c, T2, and FLAIR, to train three models for BM detection: the conventional U-Net (cU-Net), modified U-Net (moU-Net), and U-Net specifically trained for small BMs (<0.4 mL) termed sU-Net. The moU-Net incorporates loss in the decoder layers as in deep supervision. The sU-Net begins with a pre-trained U-Net and is further trained exclusively on small BMs (<0.4 mL). In their experiments, the combined model (ensemble) of the three U-Nets yielded the best performance, achieving a DSC of 0.74, sensitivity of 0.82, and a false positive rate (FPR) of 0.35, with higher sensitivity observed for larger lesions [29].

Zhou et al. utilized T1c MRI and a single-shot detector (SSD) with a 2D slice as input for BM detection. They evaluated the model’s detection performance separately on BM groups of different sizes. The sensitivities were 0.15, 0.70, and 0.98, and the positive predictive values (PPVs) were 1, 0.35, and 0.36, for BM sizes < 3 mm, between 3 and 6 mm, and greater than 6 mm, respectively. The overall sensitivity was 0.81, and the overall PPV was 0.36 [30].

The same group of authors extended their research on BM segmentation following BM detection using a larger patient dataset comprising 934 patients. They employed a segmentation network based on the 2D U-Net architecture with an input patch dimension of 64 × 64 × 3. Experimenting with a combination of focal dice and cross-entropy for the loss function, their optimal model achieved a DSC of 0.81, sensitivity of 0.85, and PPV of 0.58 across a broad range of BM sizes (1–52 mm) [31].

Zhang et al. employed 3D T1c MRI to train a 2D regional model, Faster R-CNN, for BM detection. They utilized a random under-sampling boosting strategy, referred to as RUSBoost, to enhance accuracy by elevating the utilization rate of samples incorrectly classified in the previous training for subsequent rounds. Their approach achieved sensitivity of 0.87 and an FPR of 0.24 per slice [32].

Junger et al. retrained the DeepMedic model using multi-sequence MRI including T1, T1c, T2, and T2-FLAIR images, with a patch dimension of 25 × 25 × 25. Ground truth data were obtained through manual segmentation on T1c images. The authors preprocessed the images, involving skull stripping, co-registration, bias field correction, and resampling to 1 × 1 × 1 mm^3^. Their results yielded a DSC of 0.72, sensitivity of 0.85, and an FPR of 1.5 per scan. They noted that missed detections (0.05 ± 0.04 cm^3^) were significantly smaller than detected ones (0.96 ± 2.4 cm^3^) [33].

Rudie et al. trained 3D U-Net models using either T1c or the subtraction image (T1c-T1) as input, employing a patch dimension of 96 × 96 × 96. Preprocessing included co-registration, image subtraction, resampling, and intensity normalization. The authors experimented with various combinations of loss functions, including dice and focal cross-entropy, with different weightings. They reported results using an ensemble of these variations, achieving a DSC of 0.75 and sensitivity of 0.70. Additionally, they observed a DSC of 0.85 and sensitivity of 0.88 between two manual segmentations [34].

Cao et al. modified U-Net by adopting a second down-sampling path to incorporate small kernels of 1 × 1 × 3, termed asymmetric U-Net, for BM segmentation. The authors opted to exclude cases with a single BM to focus on more challenging scenarios involving multiple lesions. The data had a resolution of 1 × 1 × 2 mm^3^. Notably, the 2 mm resolution in the third dimension may be insufficient for detecting small lesions. Various learning rates were experimented with, as some did not lead to convergence or resulted in a suboptimal model. During model testing, they separated BMs into two groups, small (1–10 mm) and large (11–26 mm). For small lesions, the DSC, sensitivity, and precision were 0.65, 0.76, and 0.72, respectively; for large lesions, they were 0.84, 0.94, and 0.82, respectively [35].

Hsu et al. utilized T1c and contrast-enhanced computed tomography (CECT) for the BM detection/segmentation task. The images were co-registered by the planner and preprocessed, which involved resampling and intensity normalization. The authors adopted a multi-stage CNN network based on V-Net to perform skull removal (brain extraction) and BM detection/segmentation. After brain extraction, they used a patch volume of 48^3^ mm^3^ as input for the BM segmentation network modified from V-Net by adding more feature maps and more layers in each block. They experimented with combinations of boundary loss and dice loss for network training and found that using 4% of boundary loss achieved the optimal results. They also found that using both T1c and CECT (sensitivity: 0.9, PPV: 0.55) resulted in fewer false positives than using T1c alone (sensitivity: 0.9, PPV: 0.45) for BM detection [36].

Liang et al. aimed to assess deep learning CNN models for automatic segmentation of BM using heterogeneous data. Their dataset comprised T1c and FLAIR images from 407 patients collected across 98 institutions. The authors adapted the U-Net architecture by modifying input dimensions, kernel numbers, normalization layers, and loss functions. Preprocessing steps included bias field correction, co-registration, resampling, Gaussian smoothing for contours, z-score intensity normalization, and brain volume extraction. Their best model, utilizing an input size of 64 × 64 × 64 × 2, achieved a DSC of 0.73, sensitivity of 0.91, and an FPR of 1.7 [37].

Ottesen et al. adopted HRNetV2, a high-resolution network that fuses high-resolution features in the encoding path, and explored two input scenarios, 2.5D and 3D, for BM segmentation. They utilized datasets from two institutions: 156 patients from their institution and 65 patients from another. Since the second dataset had fewer imaging sequences, the authors applied input layer dropout. Training involved 150 epochs with ~12,000 slices per epoch for the 2.5D model with 10× more sampling from the positive slices relative to the negative slices, and 100 epochs with 95 volumes per epoch for the 3D model. Results indicated comparable performance among 2.5D, 3D, and a baseline nnUNet [38].

Fairchild et al. utilized T1c images to train DeepMedic with an input dimension of 25 × 25 × 25. The data underwent preprocessing steps including resampling, skull stripping, and z-score intensity normalization. BMs were categorized into three groups for model evaluation: prospectively identified metastases (PIM), representing those typically manually identified; retrospectively identified metastases (RIM), representing initially missed lesions identified in later imaging; and those <3 mm in diameter. The model achieved sensitivities of 0.94, 0.8, and 0.79 for PIM, RIM, and BM < 3 mm, respectively [39].

Yu et al. devised a coarse-to-fine framework that combined central point-guided SSD, data cascade, and multi-head U-Net for BM detection and segmentation. They utilized T1c MRI with an input dimension of 128 × 128 to train SSD for 2D detection. The data cascade unit selected three consecutive slices with similar detections from SSD to form 2.5D patches for multi-head U-Net segmentation. Their models achieved sensitivity of 0.91 and a PPV of 0.77 for BMs ≤ 1.5 cc, and a DSC of 0.86 for BMs > 1.5 cc [40].

Buchner et al. employed a 3D UNet for BM segmentation, utilizing data from multiple centers. They preprocessed the data using the BraTS-Toolkit [43], involving registration, skull stripping, and alignment to the BraTS Atlas. To address missing sequences, a generative adversarial network synthesized data for patients lacking one of the required four sequences for model input. The authors reported a mean DSC of 0.92 and an F1 score of 0.93. Notably, the BM size in their dataset was generally large, with a mean volume of 13 mL [41].

## 3. Summary of Segmentation Strategies

In general, strategies in BM detection and segmentation can be categorized based on the imaging sequence used, the approach employed (regional for detection or voxel-based for segmentation), network architectures, pre- and postprocessing methods, and training and testing procedures.

### 3.1. Input Sequence

T1c MR imaging is the most common input sequence for BM detection due to higher overall sensitivity as a screening test and improved contrast compared to its contrast-enhanced CT alternative [44]. Specifically, for surgery or radiosurgery, T1c is recommended for diagnosis and treatment planning of BMs [45]. Several studies have utilized multiparametric sequences such as T1, T1c, T2, and T2 FLAIR, which are also utilized in the BraTS Challenge. These sequences are useful for identifying surrounding vasogenic edema, frequently associated with BMs [44], while the detection of enhancing lesions still predominantly relies on T1c imaging. However, the increased time required to collect the needed images and the deviation from the typical SRS clinical workflow have rendered multiparametric imaging clinically unpopular. Regardless of the imaging sequences, high-resolution images (1 × 1 × 1 mm^3^) are essential, given that BM can be as small as a few millimeters.

### 3.2. Regional and Voxel-Based Approaches

Regional approaches typically involve predicting bounding boxes over images and then classifying each box, such as determining the presence of BM. In BM detection, a regional approach might involve identifying areas of contrast enhancement in T1c MRI. Various deep learning regional proposal/detection tools, including R-CNN [46], Faster R-CNN [47], YOLO [48], YOLOv3 [49], and SSD [50], are available for this purpose. Faster R-CNN enhances R-CNN by integrating regional proposals into the network architecture. Similarly, YOLOv3 represents an advancement over YOLO by incorporating multiscale prediction, leading to an improved detection of small objects.

The predicted bounding boxes may be used for subsequent focused segmentation to mitigate computational cost in directly segment the whole volume. However, predicting bounding boxes can pose its own challenges.

On the other hand, voxel-based approaches classify each voxel, enabling fine-grained segmentation, and inherently rendering detection. Hence, while some studies stated both detection and segmentation in the title, their approaches may involve only segmentation networks. In BM segmentation, a voxel-based approach may classify each voxel as tumor or non-tumor based on intensity and spatial relationships. Fully convolutional architectures, such as DeepMedic [42] and U-Net [51], have been employed for conducting the segmentation task.

### 3.3. Network Architecture

**Figure 2:**
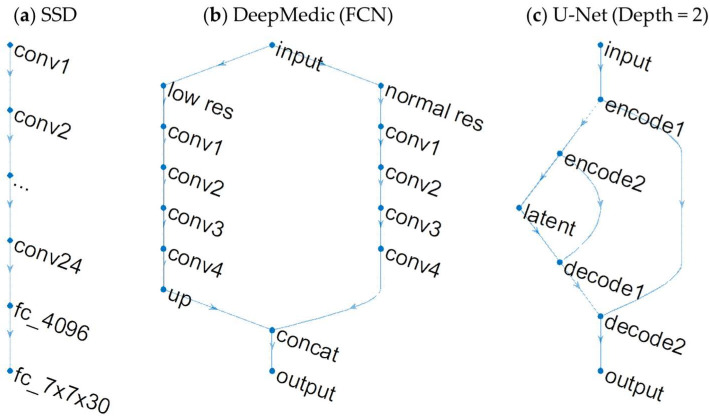
Illustration of network architectures for BM detection and segmentation. (**a**) SSD network. This example shows the dimensions of YOLO. The last layer outputs predicted bounding boxes and class probability with data dimensions of 7 × 7 × 30. (**b**) DeepMedic, an FCN type network. It combines a normal resolution and a low-resolution branch with output corresponding to the receptive fields. (**c**) U-Net. The encoder branch and decoder branch are connected at the same depth. Here, we illustrate U-Net with a depth of two.

There are three basic network architectures, SSD, FCN, and U-Net, utilized in the reviewed studies (Figure 2).

The SSD-type networks alter the high-level layers of a classification network by incorporating a fully connected layer for output, which represents predicted bounding boxes and class probabilities. CropNet (Dikici et al., 2020), SSD (Zhou et al., 2020), and Faster R-CNN with VGG16 (Zhang et al., 2020) belong to this type [26,30,32].

The FCNs either output representation of receptive fields or utilize deconvolution in the final layers for dense segmentation. DeepMedic (Kamnitas et al., 2017) [42] is a popular voxel-based 3D CNN in the first category. It has been utilized across various studies, including those by Liu et al. (2017), Charron et al. (2018), Junger et al. (2021), and Fairchild et al. (2023) [23,24,33,39]. The studies utilizing GoogLeNet as the backbone by Grovik et al. (2020) and HRNetV2 by Ottesen et al. (2022) belong to the second category [27,38].

The U-Net architecture is a prevalent choice for segmentation tasks, leveraging a residual mechanism to connect encoding and decoding levels, thereby enhancing stability and convergence. Studies employing U-Net include those by Bousabarah et al. (2020), Rudie et al. (2021), Cao et al. (2021), Hsu et al. (2021), Liang et al. (2022), Yu et al. (2023), and Buchner et al. (2023) [29,34,35,36,37,40,41].

Segmentation following detection is often accomplished through cascaded networks, as seen in BMDS (Xue et al., 2020), MetNet (Zhou et al., 2020), and DeSeg (Yu et al., 2023) [28,31,40]. Commonly applied loss functions include dice loss and cross-entropy loss, with modifications such as focal loss to address class imbalance. While 2D CNN has been extensively used for computer vision tasks on natural images, processing each 2D slice individually may lead to the loss of volumetric information when segmenting 3D MRI scans. To mitigate this, some studies employ a 2.5D approach, incorporating adjacent slices. Alternatively, 3D CNNs, while effective, can be computationally demanding. Patch-wise processing, a feature of nnU-Net [52], helps alleviate this computational burden.

### 3.4. Pre- and Postprocessing

Common preprocessing encompasses registration, resampling, skull stripping, and intensity adjustment through methods like gamma correction or z-scoring [25]. These steps aim to align brains in a common space and standardize intensity ranges for consistency and comparability to improve network learning.

Following segmentation, common postprocessing methods are employed to refine results. These may include false positive removal using techniques such as sphericity thresholding [23,53], blob filtering, fully connected conditional random fields (CRF) [42], or ensemble classifiers [54]. These postprocessing steps help enhance the accuracy and quality of segmented images by eliminating artifacts or noise.

### 3.5. Training and Testing Procedures

Data augmentation is a widely used technique in medical image segmentation, often involving adjustments to intensity, rotation, flipping, and deformation [26,36]. Some studies have even applied data augmentation during test time to further enhance model robustness. Furthermore, to tackle data imbalance, certain studies enforce balanced class sampling during training by augmenting the sampling rate for positive cases of BMs [38]. Additionally, focal loss has been employed in some studies to mitigate the effects of data imbalance. It is important to carefully balance training batch sizes to ensure adequate data representation while avoiding excessive computational burden.

## 4. Summary of Data Utilization

Most of the studies utilized single institutional data (15 out of 19 studies). The remaining studies utilized multi-institutional data from two centers (Ottesen et al. [38]), three centers (Xue et al. [28]), six centers (Buchner et al. [41]), and ninety-eight centers (Liang et al. [37]). The last source comprised data from a clinical trial [55]. The data used by a couple of studies (Grovik et al. [27] and Rudie et al. [34]) are publicly available [56,57].

Additional data attributes, such as MR field strength and dataset size, are summarized in Table 1.

## 5. Summary of Segmentation Performance

Segmentation performance is typically evaluated using the DSC, also known as the F1 score for voxel-wise prediction. This metric measures the agreement between the prediction and ground truth, normalized by their average. Consequently, DSC and its variations are commonly used to define the loss function for network optimization. However, DSC may not properly reflect detection performance for small lesions, as missing a few voxels can significantly impact the score. For small lesions, it is more appropriate to evaluate performance at the lesion level rather than at the voxel level. Therefore, sensitivity and precision can be used instead of DSC, and they define the F1 score at the lesion level. While the reviewed studies have used various sets of metrics, our focused metrics for the performance summary are DSC, detection sensitivity, and precision.

The DSC performance had a wide range, ~0.7 to 0.9, and the sensitivity also presented a wide range from ~0.6 to >0.9, among different studies, likely reflecting significant differences in the distribution of BM size studied. Furthermore, variations in evaluation criteria across studies further hinder direct comparison of model performance. For example, when true negative was assessed using the background [28], it would dominate the specificity but would not accurately reflect the performance of BM segmentation. Reporting precision or false detection rate (=1 − precision), instead of reporting false positives per patient or per slice, could provide a more standardized approach for assessing detection performance. The parameters and performance of the studies are summarized in Table 1.

## 6. Discussion

### 6.1. Needs and Challenges

The primary motivation for automatic BM detection and segmentation in a clinical setting is to enhance the accuracy and efficiency of lesion delineation. Manual delineation is both time-consuming and challenging, especially for small BMs, due to their diminutive sizes requiring finer resolutions, their resemblance in shape and contrast to surrounding blood vessels, and their low contrast against adjacent tissues [32]. Clinicians must invest time in verifying BMs across a 3D space by examining individual 2D MRI slices [32], a process prone to detection errors as clinicians lack prior knowledge of BM locations [15,58,59].

Most automated detection and segmentation continue to miss small BMs or are hindered by high false detection rates. This can be attributed to several primary factors: (1) DSC bias to large lesions; (2) complex segmentation for heterogenous BM structure; (3) data imbalance between positive and negative cases; (4) quality of input MR images that have low contrast, a lack of volumetric data, or insufficient resolution; and (5) limitation of mono-modality imaging [24].

### 6.2. Addressing the Challenges

#### 6.2.1. Train a Separate Model for Small BM

When reporting model evaluation, the combined results for small and large BMs may not reveal the performance for small BM detection [30]. Similarly, when training models to detect both small and large BMs, the small ones may be assigned less weight, making it challenging to improve detection of small lesions. Studies that assessed model performance separately for small and large BMs have shown significant disparities in results [28], suggesting the need for dedicated models.

Recent studies have begun to differentiate between CNN models tailored to either small or large BMs [60]. For example, to train a model focused on small BMs, it can be achieved by masking large BMs in the training data. However, when training a separate model specifically for small BM detection, the commonly used DSC may exhibit greater sensitivity to misalignments between ground truth and predicted voxel segmentation, potentially leading to less stable training. To mitigate this issue, optimization loss functions that address volumetric bias may improve small BM delineation, as favored in recent studies. These include techniques such as focal dice or focal cross-entropy, commonly utilized in regional approaches, which can be implemented during model training.

#### 6.2.2. Multi-Modality May Help but T1c Is More Practical and May Be Sufficient

Multi-modality images may facilitate BM detection. For example, T2 FLAIR may be used to guide attention for BM segmentation especially when there is no sufficient contrast reaching the tumor on T1c [24], while T1 arguably does not help with detection accuracy [61]. Multi-modality and cross-modality input sequences were initially investigated, but they require additional clinical implementation and increase clinical turnaround time for SRS treatment. Studies that utilized only T1c have shown performance comparable to those using parametric MRI (Table 1).

#### 6.2.3. Limit the Amount of Preprocessing

The preprocessing framework that aligns brains spatially and standardizes image intensity range can assist in identifying abnormalities and is an integral part of the processing scheme in nnU-Net. However, for BM detection, preprocessing steps involving image interpolation may reduce the detectability of small BMs.

Similarly, skull stripping is a common preprocessing step for BM segmentation [43,62]. It has been explored to enhance CNN prediction by addressing hyper-intensities related to surrounding vasculature, often present along the skull. However, this step eliminates the possibility of identifying BMs along the brain’s surface perimeter.

In general, preprocessing methods that entail image interpolation should be avoided as they could result in smearing of BMs, making small lesions more challenging to detect. While aligning the brain to an atlas can facilitate automatic standardized labeling, this step can be performed after BM segmentation.

#### 6.2.4. Network Tricks: Loss Function, Deep Supervision, Patch-Wise Training to Increase Training Data, Weighted Sampling, and Ensemble

In general, the U-Net type architecture has demonstrated great performance in segmentation task [52]. Yet, there are potential modifications that could enhance BM segmentation. For instance, focal loss can be employed to address data imbalances [38], and larger weights can be applied to incorrectly classified cases [63]. Altering loss function terms and weights can significantly impact image detection and classification tasks [48]. Deep supervision, a technique that has been applied for brain tumor segmentation [64], and patch-wise training, which has the effect of increasing training data [65], are among the strategies that can be utilized.

Additionally, common approaches such as data augmentation and balancing training sample classes have been employed to enhance model performance [26]. Another effective strategy involves using an ensemble of models trained on different datasets, akin to an ensemble of k models in k-fold training.

#### 6.2.5. Benchmarking: Evaluation Metrics, Public Data, and Competition/Challenges

**Figure 3:**
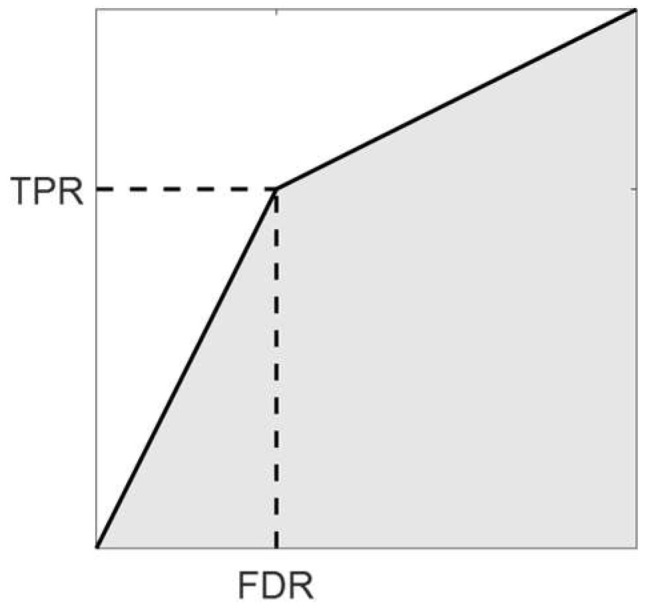
The area under curve (AUC, the shaded region) for the true positive rate (TPR) against false detection rate (FDR) can be approximated as the average of TPR and PPV at an instance of detection threshold.

Ensuring standardized segmentation metrics and reporting results consistently across studies can facilitate comparisons and benchmarking efforts. For example, specificity is not a meaningful measure for BM detection since true negatives (TNs) are dominant when most voxels are non-tumor voxels (negative), and positive detection has a small fraction. The ratio tends to be large even for poor detection accuracy. Reporting detection accuracy in terms of sensitivity (recall) and precision (PPV), which equals one minus the false detection rate, can better illuminate the model performance and make comparison easier between studies. In addition, the average of sensitivity and positive prediction value has the equivalence of measuring the area under curve (AUC) for the curve plotted for the true positive rate (TPR) against false detection rate (FDR). This can be illustrated as follows (Figure 3).

$$\begin{array}{c} TPR=\frac{TP}{TP+FN},\;PPV=\frac{TP}{TP+FP},\;FDR=\frac{FP}{TP+FP}=1-PPV\\ AUC=\frac{1}{2}FDR\cdot TPR+\frac{1}{2}\left(TPR+1\right)\cdot \left(1-FDR\right)=\frac{1}{2}\left(TPR+PPV\right) \end{array}$$

The BraTS Challenge exemplifies a public domain for testing and benchmarking models. BM segmentation was as a part of a recent challenge [20]. The organizers trained annotators with MR physics and image intensity features to label data. It could be helpful to make the training material available to the research community. The BraTS BM Challenge prepared data with manual annotation, requiring processing time ranging from several minutes to hours for a single case, depending on the number of BM in a case [20].

The BM segmentation challenge provided four MR sequences, T1, T1c, T2, and FLAIR, as many other tumor segmentation challenges. The challenge entailed segmentation of edema, which often encircles larger lesions. T1c, commonly used clinically, may offer greater sensitivity for algorithms compared to other sequences [61]. However, there are different sequences for T1c, such as 2D spin echo (2D-SE), 3D MP-RAGE, SPACE, black blood sequences, etc., and some may be more suitable than others for BM detection [66,67,68,69]. Subtraction images of T1c and T1 may not aid in detecting small BMs. They might end up highlighting blood vessels, which become harder to distinguish from BM when the background is removed. The hyperintense blood vessels are typically seen between cerebrospinal fluid (CSF) and the surface of cortex when the blood vessels ramify on the surface of cortex, while small metastases are often seen between the gray and white matter junctions. But this may not be a clear distinction since the gray matter folds and the blood vessel on the cortex may appear to be at junctions. This problem only concerns small metastases. Another feature that can be used to distinguish blood vessels from BMs is that the blood vessels are continuous while small lesions are typically isolated and only show in a few slices. Three-dimensional sequencing with 1 mm slice resolution is needed for BM < 5 mm [66,67].

### 6.3. Prospective Ongoing Strategy

Segmentation results provide essential diagnostic insights, are crucial for planning, and facilitate response evaluation. Automatic BM segmentation aims to enhance efficiency and accuracy, while also unlocking benefits such as enabling consistent tumor volume measurements for reliable response assessment [70]. Additionally, it allows the summarization of results in terms of MR image features like VASARI [71], with summary statistics serving as feedback to improve segmentation, whether through manual annotation or machine learning. Integrating clinical data with features and statistics can further refine models for improved BM segmentation.

Current gaps in research on small BM segmentation include a need for clinical validation of CNN strategies for prospective patients or overall implementation of automated tools within the SRS workflow. Clinical application of the state-of-the-art automatic BM detection and segmentation can serve as an assistive function by providing initial detection, segmentation, and labeling. This helps segmentation efficiency and accuracy and fosters segmentation consistency. Clinical application could extend to employing CNN models for follow-up SRS treatments and identifying newly formed BMs across longitudinal images datasets. Leveraging clinical knowledge, medical history, and patient presentation can provide valuable insights into BM detection and segmentation. For instance, metastases often follow the blood route to the brain and tend to settle at the junction between gray and white matter and at border zones between major arteries [66]. Additionally, the primary disease and disease state may offer indications about BM features; for example, metastases from certain primary diseases like melanoma are more prone to hemorrhage; and metastases from lung cancer tend to manifest as multiple lesions [66].

These factors underscore the importance of integrating automatic BM segmentation into clinical practice. While initial segmentation results may require clinician review and modification for SRS planning and treatment, strategies that augment clinician identification of BMs rather than fully automate BM delineation can still prove clinically valuable. For instance, providing clinicians with regional probability estimates may expedite workflow and reduce missed or incorrect BM identification.

To harness the benefits of automatic segmentation, we have seamlessly integrated our tools into clinical practice [53]. These tools offer initial segmentation results that clinicians can readily incorporate into their workflow, serving as semi-automatic aids. Furthermore, the tools have the capability to automatically generate lesion labels based on customizable standardization or individual physician requirements, resulting in significant time savings. Through this integration, we have been able to continuously evaluate our models and implement improvements based on user feedback. For instance, both our own experience and findings from various studies suggest that ensemble approaches employing U-Net-like structures trained on diverse datasets yield substantial enhancements in performance. Developing lesion size-specific models may represent a promising avenue for addressing the challenges associated with small BM segmentation.

## 7. Conclusions

Recent advances in CNN have presented promising opportunities for developing automatic BM segmentation tools. Overcoming the challenges associated with segmenting small BMs may necessitate the development of dedicated models tailored to this task. Standardizing segmentation metrics and reporting practices enable effective benchmarking of segmentation algorithms. Integrating segmentation tools into clinical workflows, where they can function as semi-automatic segmentation aids and offer automatic labeling, is essential for enhancing the accuracy and efficiency of BM detection and segmentation. Moreover, continuous model evaluation based on user feedback is critical for ensuring ongoing improvements in performance and clinical utility.

## Figures and Tables

**Table 1:** Summary of the reviewed studies. For brevity, C# denotes the column number. (C1) The study number. (C2) The first author. (C3) The year of publication. (C4) The task. Det denotes detection, and Seg denotes segmentation. If the study explicitly implements detection and segmentation in two steps, it is denoted as Det+Seg. (C5) The image sequences used in the study. (C6) MR field strength. (C7) The CNN model of BM detection/segmentation. (C8) The network (kernel) dimension. (C9) The input data dimension. (C10) The optimization loss functions. (C11) The training, validation, and testing setup. (C12) The BM size. (C13) Performance. “DSC”: dice similarity coefficient. “Sen”: sensitivity. “FPR”: false positive rate. “PPV”: positive prediction value, or precision. “Spe”: specificity.

No	Study	Year	Task	Sequence	Field Strength	CNN Model	Network Dimension	Input	Optimization Loss	Train/Val/Test	BM Size	Performance
1	Liu et al. [23]	2017	Seg	T1c	3T	En-DeepMedic	3D	Patch 25 × 25 × 25	CE	5-fold	mean 670 mm^3^	DSC 0.67
2	Charron et al. [24]	2018	Seg	T1, T1c, FLAIR	1.5T	DeepMedic	3D	Patch 24 × 24 × 24	DICE	146/18/18	median 70 mm	DSC 0.78, Sen 0.97, FPR 5.9 per patient
3	Hu et al. [25]	2019	Seg	T1c, CT	Unspecified	UNet+DeepMedic	3D	512 × 512 × 8 × 2 for U-Net	Focal DICE	245/30/76 patients	median 760 mm^3^	<1500 mm^3^: DSC 0.47, Sen 0.61; >1500 mm^3^: DSC 0.82, Sen 0.98
4	Dikici et al. [26]	2020	Det	T1c	1.5T, 3T	CropNet	3D	Patch 16 × 16 × 16	CE	5-fold (217 scans, 158 patients)	mean: 5.4 mm; 160 mm^3^	Sen 0.9; FPR 9.12 per patient
5	Grøvik et al. [27]	2020	Seg	T1-BRAVO, T1, T1c, and FLAIR	1.5T, 3T	GoogLeNet	2.5D	256 × 256 × 28	CE	100/5/51	mode ~10 mm	DSC 0.79, Sen 0.53, PPV 0.79
6	Xue et al. [28]	2020	Det+Seg	T1c	3T	BMDS (cascaded FCN)	3D	256 × 256 × 120	DICE	4-fold	median 16 mm	DSC 0.85, Sen 0.96, Spe 0.99
7	Bousabarah et al. [29]	2020	Seg	T1c, T2, and FLAIR	3T	U-Net, moU-Net, sU-Net	3D	Patch 128 × 128 × 128	DICE	469/0/40	median 470 mm^3^	DSC 0.74, Sen 0.82, FPR 0.35
8	Zhou et al. [30]	2020	Det	T1c	1.5T, 3T	Single Shot Dector	2D	256 × 256 × 1	Detector	212/0/54(234) patients (BM)	mean 10 mm	Sen 0.81, PPV 0.36
9	Zhou et al. [31]	2020	Det+Seg	T1c	1.5T, 3T	MetNet	2D	Patch 64 × 64 × 3	Focal DICE	748/0/186	mode 3–6 mm	DSC 0.81, Sen 0.85, PPV 0.58
10	Zhang et al. [32]	2020	Det	T1c	1.5T, 3T	Faster R-CNN + RUSBoost	2D	256 × 256 × 1	CE + L1	270/0/91 scans	unspecified	Sen 0.87, FPR 0.24 per slice
11	Junger et al. [33]	2021	Seg	T1, T1c, T2, and FLAIR	1T, 1.5T, 3T	DeepMedic	3D	Patch 25 × 25 × 25	DICE	66(248)/0/17(67) patients (BM)	mean 990 mm^3^	DSC 0.72, Sen 0.85, FPR 1.5 per scan
12	Rudie et al. [34]	2021	Seg	T1c or T1c-T1	1.5T, 3T	3D U-Net	3D	Patch 96 × 96 × 96	DICE + focal CE	413/50/100 scans	median 50 mm^3^	DSC 0.75, Sen 0.7, FPR 0.46 per scan
13	Cao et al. [35]	2021	Seg	T1c	1.5T	asym-UNet	3D	256 × 256 × 80	CE	160(809)/20(136)/15(89) patients (BM)	mode 3.5 mm	<10 mm: DSC 0.65, Sen 0.76, PPV 0.72; >11 mm: DSC 0.84, Sen 0.94, PPV 0.82
14	Hsu et al. [36]	2021	Seg	T1c and CECT	1.5T, 3T	V-net	3D	Patch 48 × 48 × 48	boundary loss + DICE	(402, 5-fold)/102	mode 7.5 mm	DSC 0.76, Sen 0.9, FPR 2.4
15	Liang et al. [37]	2022	Seg	T1c and FLAIR	Unspecified	U-Net (variant)	3D	Patch 64 × 64 × 64 × 2	DICE	326 (78)/0/81 (20) patients (centers)	median 17.6 mm	DSC 0.73, Sen 0.91, FPR 1.9 per patient
16	Ottesen et al. [38]	2022	Seg	T1, T1c, FLAIR, BRAVO (Set II)	Unspecified	HRNetV2	2.5D/3D	Unspecified	Focal +CE	160/10/51	unspecified	Sen 0.79, FPR 6.2 per patient/Sen 0.71, FPR 3.2
17	Fairchild et al. [39]	2023	Seg	T1c	1.5T, 3T	DeepMedic+	3D	Patch 25 × 25 × 25	DICE	4-fold	median 5.6 mm	DSC 0.79, Sen 0.89, PPV 0.59
18	Yu et al. [40]	2023	Det+Seg	T1c	1.5T, 3T	DeSeg (U-net)	2+2.5D	256 × 256 × 1	Focal + CE	192/24/24 patients	median < 50 mm^3^	DSC 0.86, Sen 0.91, PPV 0.77
19	Buchner et al. [41]	2023	Seg	T1, T1c, T2, and FLAIR	Unspecified	U-Net	3D	192 × 192 × 32	DICE + CE	260/0/88 patients	median 13,000 mm^3^	DSC 0.92, F1 0.93

## References

[1] Achrol A.S., Rennert R.C., Anders C., Soffietti R., Ahluwalia M.S., Nayak L., Peters S., Arvold N.D., Harsh G.R., Steeg P.S. (2019). Brain metastases. Nat. Rev. Dis. Primers.

[2] Arvold N.D., Lee E.Q., Mehta M.P., Margolin K., Alexander B.M., Lin N.U., Anders C.K., Soffietti R., Camidge D.R., Vogelbaum M.A. (2016). Updates in the management of brain metastases. Neuro-Oncol..

[3] Graber J.J., Cobbs C.S., Olson J.J. (2019). Congress of Neurological Surgeons Systematic Review and Evidence-Based Guidelines on the Use of Stereotactic Radiosurgery in the Treatment of Adults with Metastatic Brain Tumors. Neurosurgery.

[4] Lam T.C., Sahgal A., Chang E.L., Lo S.S. (2014). Stereotactic radiosurgery for multiple brain metastases. Expert Rev. Anticancer Ther..

[5] Park H.S., Chiang V.L., Knisely J.P., Raldow A.C., Yu J.B. (2011). Stereotactic radiosurgery with or without whole-brain radiotherapy for brain metastases: An update. Expert Rev. Anticancer Ther..

[6] Quigley M.R., Fuhrer R., Karlovits S., Karlovits B., Johnson M. (2008). Single session stereotactic radiosurgery boost to the post-operative site in lieu of whole brain radiation in metastatic brain disease. J. Neuro-Oncol..

[7] Lippitz B., Lindquist C., Paddick I., Peterson D., O’Neill K., Beaney R. (2014). Stereotactic radiosurgery in the treatment of brain metastases: The current evidence. Cancer Treat. Rev..

[8] Soliman H., Das S., Larson D.A., Sahgal A. (2016). Stereotactic radiosurgery (SRS) in the modern management of patients with brain metastases. Oncotarget.

[9] Nieder C., Grosu A.L., Gaspar L.E. (2014). Stereotactic radiosurgery (SRS) for brain metastases: A systematic review. Radiat. Oncol..

[10] Chang A.T.Y., Tan L.T., Duke S., Ng W.T. (2017). Challenges for Quality Assurance of Target Volume Delineation in Clinical Trials. Front. Oncol..

[11] Growcott S., Dembrey T., Patel R., Eaton D., Cameron A. (2020). Inter-Observer Variability in Target Volume Delineations of Benign and Metastatic Brain Tumours for Stereotactic Radiosurgery: Results of a National Quality Assurance Programme. Clin. Oncol..

[12] Ambrosini R.D., Wang P., O’Dell W.G. (2010). Computer-aided detection of metastatic brain tumors using automated three-dimensional template matching. J. Magn. Reson. Imaging.

[13] Sunwoo L., Kim Y.J., Choi S.H., Kim K.G., Kang J.H., Kang Y., Bae Y.J., Yoo R.E., Kim J., Lee K.J. (2017). Computer-aided detection of brain metastasis on 3D MR imaging: Observer performance study. PLoS ONE.

[14] Bauer S., Nolte L.P., Reyes M. (2011). Fully automatic segmentation of brain tumor images using support vector machine classification in combination with hierarchical conditional random field regularization. Medical Image Computing and Computer-Assisted Intervention–MICCAI 2011: 14th International Conference, Toronto, ON, Canada, September 18–22, 2011.

[15] Farjam R., Parmar H.A., Noll D.C., Tsien C.I., Cao Y. (2012). An approach for computer-aided detection of brain metastases in post-Gd T1-W MRI. Magn. Reson. Imaging.

[16] Liu Y., Stojadinovic S., Hrycushko B., Wardak Z., Lu W., Yan Y., Jiang S.B., Timmerman R., Abdulrahman R., Nedzi L. (2016). Automatic metastatic brain tumor segmentation for stereotactic radiosurgery applications. Phys. Med. Biol..

[17] Cho S.J., Sunwoo L., Baik S.H., Bae Y.J., Choi B.S., Kim J.H. (2021). Brain metastasis detection using machine learning: A systematic review and meta-analysis. Neuro-Oncol..

[18] Havaei M., Guizard N., Larochelle H., Jodoin P.M. (2016). Deep Learning Trends for Focal Brain Pathology Segmentation in MRI. Machine Learning for Health Informatics: State-of-the-Art and Future Challenges.

[19] Ozkara B.B., Chen M.M., Federau C., Karabacak M., Briere T.M., Li J., Wintermark M. (2023). Deep Learning for Detecting Brain Metastases on MRI: A Systematic Review and Meta-Analysis. Cancers.

[20] Moawad A.W., Janas A., Baid U., Ramakrishnan D., Jekel L., Krantchev K., Moy H., Saluja R., Osenberg K., Wilms K. (2023). The Brain Tumor Segmentation (BraTS-METS) Challenge 2023: Brain Metastasis Segmentation on Pre-treatment MRI. arXiv.

[21] Grishchuk D., Dimitriadis A., Sahgal A., De Salles A., Fariselli L., Kotecha R., Levivier M., Ma L., Pollock B.E., Regis J. (2023). ISRS Technical Guidelines for Stereotactic Radiosurgery: Treatment of Small Brain Metastases (</=1 cm in Diameter). Pract. Radiat. Oncol..

[22] NICE (2021). Brain Tumours (Primary) and Brain Metastases in Adults.

[23] Liu Y., Stojadinovic S., Hrycushko B., Wardak Z., Lau S., Lu W., Yan Y., Jiang S.B., Zhen X., Timmerman R. (2017). A deep convolutional neural network-based automatic delineation strategy for multiple brain metastases stereotactic radiosurgery. PLoS ONE.

[24] Charron O., Lallement A., Jarnet D., Noblet V., Clavier J.B., Meyer P. (2018). Automatic detection and segmentation of brain metastases on multimodal MR images with a deep convolutional neural network. Comput. Biol. Med..

[25] Hu S.-Y., Weng W.-H., Lu S.-L., Cheng Y.-H., Xiao F., Hsu F.-M., Lu J.-T. (2019). Multimodal Volume-Aware Detection and Segmentation for Brain Metastases Radiosurgery. arXiv.

[26] Dikici E., Ryu J.L., Demirer M., Bigelow M., White R.D., Slone W., Erdal B.S., Prevedello L.M. (2020). Automated Brain Metastases Detection Framework for T1-Weighted Contrast-Enhanced 3D MRI. IEEE J. Biomed. Health Inform..

[27] Grovik E., Yi D., Iv M., Tong E., Rubin D., Zaharchuk G. (2020). Deep learning enables automatic detection and segmentation of brain metastases on multisequence MRI. J. Magn. Reson. Imaging.

[28] Xue J., Wang B., Ming Y., Liu X., Jiang Z., Wang C., Liu X., Chen L., Qu J., Xu S. (2020). Deep learning-based detection and segmentation-assisted management of brain metastases. Neuro-Oncol..

[29] Bousabarah K., Ruge M., Brand J.S., Hoevels M., Ruess D., Borggrefe J., Grosse Hokamp N., Visser-Vandewalle V., Maintz D., Treuer H. (2020). Deep convolutional neural networks for automated segmentation of brain metastases trained on clinical data. Radiat. Oncol..

[30] Zhou Z., Sanders J.W., Johnson J.M., Gule-Monroe M.K., Chen M.M., Briere T.M., Wang Y., Son J.B., Pagel M.D., Li J. (2020). Computer-aided Detection of Brain Metastases in T1-weighted MRI for Stereotactic Radiosurgery Using Deep Learning Single-Shot Detectors. Radiology.

[31] Zhou Z., Sanders J.W., Johnson J.M., Gule-Monroe M., Chen M., Briere T.M., Wang Y., Son J.B., Pagel M.D., Ma J. (2020). MetNet: Computer-aided segmentation of brain metastases in post-contrast T1-weighted magnetic resonance imaging. Radiother. Oncol..

[32] Zhang M., Young G.S., Chen H., Li J., Qin L., McFaline-Figueroa J.R., Reardon D.A., Cao X., Wu X., Xu X. (2020). Deep-Learning Detection of Cancer Metastases to the Brain on MRI. J. Magn. Reson. Imaging.

[33] Junger S.T., Hoyer U.C.I., Schaufler D., Laukamp K.R., Goertz L., Thiele F., Grunz J.P., Schlamann M., Perkuhn M., Kabbasch C. (2021). Fully Automated MR Detection and Segmentation of Brain Metastases in Non-small Cell Lung Cancer Using Deep Learning. J. Magn. Reson. Imaging.

[34] Rudie J.D., Weiss D.A., Colby J.B., Rauschecker A.M., Laguna B., Braunstein S., Sugrue L.P., Hess C.P., Villanueva-Meyer J.E. (2021). Three-dimensional U-Net Convolutional Neural Network for Detection and Segmentation of Intracranial Metastases. Radiol. Artif. Intell..

[35] Cao Y., Vassantachart A., Ye J.C., Yu C., Ruan D., Sheng K., Lao Y., Shen Z.L., Balik S., Bian S. (2021). Automatic detection and segmentation of multiple brain metastases on magnetic resonance image using asymmetric UNet architecture. Phys. Med. Biol..

[36] Hsu D.G., Ballangrud A., Shamseddine A., Deasy J.O., Veeraraghavan H., Cervino L., Beal K., Aristophanous M. (2021). Automatic segmentation of brain metastases using T1 magnetic resonance and computed tomography images. Phys. Med. Biol..

[37] Liang Y., Lee K., Bovi J.A., Palmer J.D., Brown P.D., Gondi V., Tome W.A., Benzinger T.L.S., Mehta M.P., Li X.A. (2022). Deep Learning-Based Automatic Detection of Brain Metastases in Heterogenous Multi-Institutional Magnetic Resonance Imaging Sets: An Exploratory Analysis of NRG-CC001. Int. J. Radiat. Oncol. Biol. Phys..

[38] Ottesen J.A., Yi D., Tong E., Iv M., Latysheva A., Saxhaug C., Jacobsen K.D., Helland A., Emblem K.E., Rubin D.L. (2022). 2.5D and 3D segmentation of brain metastases with deep learning on multinational MRI data. Front. Neuroinform.

[39] Fairchild A.T., Salama J.K., Wiggins W.F., Ackerson B.G., Fecci P.E., Kirkpatrick J.P., Floyd S.R., Godfrey D.J. (2023). A Deep Learning-Based Computer Aided Detection (CAD) System for Difficult-to-Detect Brain Metastases. Int. J. Radiat. Oncol. Biol. Phys..

[40] Yu H., Zhang Z., Xia W., Liu Y., Liu L., Luo W., Zhou J., Zhang Y. (2023). DeSeg: Auto detector-based segmentation for brain metastases. Phys. Med. Biol..

[41] Buchner J.A., Kofler F., Etzel L., Mayinger M., Christ S.M., Brunner T.B., Wittig A., Menze B., Zimmer C., Meyer B. (2023). Development and external validation of an MRI-based neural network for brain metastasis segmentation in the AURORA multicenter study. Radiother. Oncol..

[42] Kamnitsas K., Ledig C., Newcombe V.F.J., Simpson J.P., Kane A.D., Menon D.K., Rueckert D., Glocker B. (2017). Efficient multi-scale 3D CNN with fully connected CRF for accurate brain lesion segmentation. Med. Image Anal..

[43] Kofler F., Berger C., Waldmannstetter D., Lipkova J., Ezhov I., Tetteh G., Kirschke J., Zimmer C., Wiestler B., Menze B.H. (2020). BraTS Toolkit: Translating BraTS Brain Tumor Segmentation Algorithms into Clinical and Scientific Practice. Front. Neurosci..

[44] Pope W.B. (2018). Brain metastases: Neuroimaging. Handb. Clin. Neurol..

[45] Soffietti R., Cornu P., Delattre J.Y., Grant R., Graus F., Grisold W., Heimans J., Hildebrand J., Hoskin P., Kalljo M. (2006). EFNS Guidelines on diagnosis and treatment of brain metastases: Report of an EFNS Task Force. Eur. J. Neurol..

[46] Girshick R., Donahue J., Darrell T., Malik J. Rich feature hierarchies for accurate object detection and semantic segmentation. Proceedings of the IEEE Conference on Computer Vision and Pattern Recognition.

[47] Ren S.Q., He K.M., Girshick R., Sun J. (2015). Faster R-CNN: Towards Real-Time Object Detection with Region Proposal Networks. arXiv.

[48] Redmon J., Divvala S., Girshick R., Farhadi A. You Only Look Once: Unified, Real-Time Object Detection. Proceedings of the 2016 IEEE Conference on Computer Vision and Pattern Recognition (CVPR).

[49] Redmon J., Farhadi A. (2018). YOLOv3: An Incremental Improvement. arXiv.

[50] Liu W., Anguelov D., Erhan D., Szegedy C., Reed S., Fu C.Y., Berg A.C. (2016). SSD: Single Shot MultiBox Detector. Proceedings of the Computer Vision–ECCV 2016: 14th European Conference.

[51] Ronneberger O., Fischer P., Brox T. U-Net: Convolutional Networks for Biomedical Image Segmentation. Proceedings of the Medical Image Computing and Computer-Assisted Intervention–MICCAI 2015: 18th International Conference.

[52] Isensee F., Jaeger P.F., Kohl S.A.A., Petersen J., Maier-Hein K.H. (2021). nnU-Net: A self-configuring method for deep learning-based biomedical image segmentation. Nat. Methods.

[53] Yang Z., Liu H., Liu Y., Stojadinovic S., Timmerman R., Nedzi L., Dan T., Wardak Z., Lu W., Gu X. (2020). A web-based brain metastases segmentation and labeling platform for stereotactic radiosurgery. Med. Phys..

[54] Yang Z., Chen M., Kazemimoghadam M., Ma L., Stojadinovic S., Timmerman R., Dan T., Wardak Z., Lu W., Gu X. (2022). Deep-learning and radiomics ensemble classifier for false positive reduction in brain metastases segmentation. Phys. Med. Biol..

[55] Brown P.D., Gondi V., Pugh S., Tome W.A., Wefel J.S., Armstrong T.S., Bovi J.A., Robinson C., Konski A., Khuntia D. (2020). Hippocampal Avoidance During Whole-Brain Radiotherapy Plus Memantine for Patients with Brain Metastases: Phase III Trial NRG Oncology CC001. J. Clin. Oncol..

[56] Rudie J.D., Saluja R., Weiss D.A., Nedelec P., Calabrese E., Colby J.B., Laguna B., Mongan J., Braunstein S., Hess C.P. (2023). The University of California San Francisco, Brain Metastases Stereotactic Radiosurgery (UCSF-BMSR) MRI Dataset. arXiv.

[57] BrainMetShare. https://aimi.stanford.edu/brainmetshare.

[58] Fairchild A., Salama J.K., Godfrey D.J., Wiggins W., Ackerson B., Niedzwiecki D., Fecci P., Kirkpatrick J.P., Floyd S.R. (2022). Early Imaging Characteristics Associated with Development of Future Brain Metastases in Patients Undergoing Stereotactic Radiosurgery. Int. J. Radiat. Oncol..

[59] Togao O., Hiwatashi A., Yamashita K., Kikuchi K., Yoshiura T., Honda H. (2014). Additional MR contrast dosage for radiologists’ diagnostic performance in detecting brain metastases: A systematic observer study at 3 T. Jpn. J. Radiol..

[60] Erdur A.C., Scholz D., Buchner J.A., Combs S.E., Rueckert D., Peeken J.C. (2023). All Sizes Matter: Improving Volumetric Brain Segmentation on Small Lesions. arXiv.

[61] Buchner J.A., Peeken J.C., Etzel L., Ezhov I., Mayinger M., Christ S.M., Brunner T.B., Wittig A., Menze B.H., Zimmer C. (2023). Identifying core MRI sequences for reliable automatic brain metastasis segmentation. Radiother. Oncol..

[62] Isensee F., Schell M., Pflueger I., Brugnara G., Bonekamp D., Neuberger U., Wick A., Schlemmer H.P., Heiland S., Wick W. (2019). Automated brain extraction of multisequence MRI using artificial neural networks. Hum. Brain Mapp..

[63] Lin T.Y., Goyal P., Girshick R., He K.M., Dollár P. (2020). Focal Loss for Dense Object Detection. IEEE Trans. Pattern Anal. Mach. Intell..

[64] Futrega M., Milesi A., Marcinkiewicz M., Ribalta P. (2022). Optimized U-Net for Brain Tumor Segmentation. Brainlesion: Glioma, Multiple Sclerosis, Stroke and Traumatic Brain Injuries. Brainles 2021.

[65] Havaei M., Davy A., Warde-Farley D., Biard A., Courville A., Bengio Y., Pal C., Jodoin P.M., Larochelle H. (2017). Brain tumor segmentation with Deep Neural Networks. Med. Image Anal..

[66] Fink K.R., Fink J.R. (2013). Imaging of brain metastases. Surg. Neurol. Int..

[67] Suh C.H., Jung S.C., Kim K.W., Pyo J. (2016). The detectability of brain metastases using contrast-enhanced spin-echo or gradient-echo images: A systematic review and meta-analysis. J. Neuro-Oncol..

[68] Komada T., Naganawa S., Ogawa H., Matsushima M., Kubota S., Kawai H., Fukatsu H., Ikeda M., Kawamura M., Sakurai Y. (2008). Contrast-enhanced MR imaging of metastatic brain tumor at 3 tesla: Utility of T(1)-weighted SPACE compared with 2D spin echo and 3D gradient echo sequence. Magn. Reson. Med. Sci..

[69] Kottlors J., Geissen S., Jendreizik H., Hokamp N.G., Fervers P., Pennig L., Laukamp K., Kabbasch C., Maintz D., Schlamann M. (2021). Contrast-Enhanced Black Blood MRI Sequence Is Superior to Conventional T1 Sequence in Automated Detection of Brain Metastases by Convolutional Neural Networks. Diagnostics.

[70] Kaufmann T.J., Smits M., Boxerman J., Huang R., Barboriak D.P., Weller M., Chung C., Tsien C., Brown P.D., Shankar L. (2020). Consensus recommendations for a standardized brain tumor imaging protocol for clinical trials in brain metastases. Neuro-Oncol..

[71] Gaillard F., Lokhande D., Jha P. (2016). VASARI MRI Feature Set. https://radiopaedia.org/articles/45816.

